# Prepackaged central line kits reduce procedural mistakes during central line insertion: a randomized controlled prospective trial

**DOI:** 10.1186/1472-6920-13-60

**Published:** 2013-04-30

**Authors:** Yelena Fenik, Nora Celebi, Robert Wagner, Christoph Nikendei, Frederike Lund, Stephan Zipfel, Reimer Riessen, Peter Weyrich

**Affiliations:** 1 University of Tuebingen Medical School, Tuebingen, Germany; 2 Department of Endocrinology, Diabetology, Nephrology, Angiology and Clinical Chemistry, University Hospital of Tuebingen, Otfried-Mueller-Str. 10, Tuebingen, 72076, Germany; 3 Department of General Internal and Psychosomatic Medicine, University Hospital of Heidelberg, Im Neuenheimer Feld 410, Heidelberg, 69120, Germany; 4 Department of Psychosomatic Medicine, University Hospital of Tuebingen, Otfried-Mueller-Str. 10, Tuebingen, 72076, Germany; 5 Medical Intensive Care Unit, University Hospital of Tuebingen, Otfried-Mueller-Str. 10, Tuebingen, 72076, Germany

**Keywords:** Central catheter placement, Cognitive load, Split-attention principle

## Abstract

**Background:**

Central line catheter insertion is a complex procedure with a high cognitive load for novices. Providing a prepackaged all-inclusive kit is a simple measure that may reduce the cognitive load. We assessed whether the use of prepackaged all-inclusive central line insertion kits reduces procedural mistakes during central line catheter insertion by novices.

**Methods:**

Thirty final year medical students and recently qualified physicians were randomized into two equal groups. One group used a prepackaged all-inclusive kit and the other used a standard kit containing only the central vein catheter and all other separately packaged components provided in a materials cart. The procedure was videotaped and analyzed by two blinded raters using a checklist. Both groups performed central line catheter insertion on a manikin, assisted by nursing students.

**Results:**

The prepackaged kit group outperformed the standard kit group in four of the five quality indicators: procedure duration (26:26 ± 3:50 min vs. 31:27 ± 5:57 min, p = .01); major technical mistakes (3.1 ± 1.4 vs. 4.8 ± 2.6, p = .03); minor technical mistakes (5.2 ± 1.7 vs. 8.0 ± 3.2, p = .01); and correct steps (83 ± 5% vs. 75 ± 11%, p = .02). The difference for breaches of aseptic technique (1.2 ± 0.8 vs. 3.0 ± 3.6, p = .06) was not statistically significant.

**Conclusions:**

Prepackaged all-inclusive kits for novices improved the procedure quality and saved staff time resources in a controlled simulation environment. Future studies are needed to address whether central line kits also improve patient safety in hospital settings.

## Background

A central line catheter is a central venous catheter typically used in critical care and oncological patients [1]. Although often vital in a clinical setting, central line catheters are associated with a number of serious complications, estimated at approximately 5.3 complications per 1000 catheter days [1,2]. Complications include catheter-related infections and mechanical complications during insertion, such as arterial puncture, catheter misplacement, pneumothorax, hemothorax, pericardial tamponade, air emboli, and even death. Late complications, including venous thrombosis, embolism and venous stenosis can also occur [3-8]. Self-limiting cardiac arrhythmias are common and are caused by irritation of the ventricle by the guide wire. Some authors have claimed that a very low complication rate is achievable for central line catheter insertions [9,10]. There is strong evidence that infectious complications can be prevented with thorough training in central line catheter placement; maximum barrier precautions; disinfection of the insertion site; hand hygiene; antiseptic coating of the catheter; timely removal of unnecessary catheters; and the use of all-inclusive catheter carts [11,12]. Failed puncture can often be avoided using ultrasound guidance [13,14].

Studies have shown that inexperienced physicians are more likely to fail or induce mechanical complications while placing a central line than their experienced colleagues [15,16]. Mechanical complications usually arise from the complex multistep insertion procedure, and identifying practices that contribute to a technically correct central line catheter insertion minimizes these complications.

One of the simplest measures to prevent central line catheter complications is using a prepackaged all-inclusive central line catheter insertion kit. Central line catheter insertion is a complex task with a high cognitive load for novices. Cognitive load theory assumes that the human cognitive system has a limited working memory of no more than five to nine information elements and actively processes no more than two to four elements simultaneously [17]. Tasks with high element interactivity are difficult to understand and produce a high cognitive load because learners must deal with several elements simultaneously [17]. There are several ways to reduce the cognitive load in novices. The split-attention principle focuses on replacing multiple sources of information, distributed either in space (spatial split-attention) or time (temporal split-attention), with one integrated source of information [17]. Placing all of the materials required for a certain procedure into one prepackaged kit represents this principle, and may facilitate the complex insertion procedure for novices. To our knowledge, there are only two studies assessing the introduction of an all-inclusive central line kit at two different intensive care units [10,18]. In both studies, several changes were introduced simultaneously (checklists, staff education, and daily central line assessments). Neither study assessed the effect of prepackaged all-inclusive central line catheter kits in reducing mechanical complications or time resources. In addition, there was no differentiation between novices and experts.

We investigated whether the use of a prepackaged all-inclusive central line catheter insertion kit by novices effectively reduces the number of procedural mistakes, procedure duration, breaches of aseptic technique, and improves adherence to the procedural algorithm.

## Methods

### Study design

This was a randomized, controlled, prospective, single-blind study to assess whether the use of a prepackaged all-inclusive central line catheter insertion kit (prepackaged kit) containing all of the necessary materials for insertion from preparation to cleanup vs. a central line catheter kit containing only the catheter components and all other separately packaged components provided in a materials cart (standard kit, Table 1) would result in fewer procedural mistakes and possibly improve asepsis breaches during the insertion procedure when performed by novices.

**Table 1:** Contents of the prepackaged and standard kits

	**Prepackaged kit**	**Standard kit**
Sterile covering	Drape 75x90 cm	
	Gown XL	
	Fenestrated drape 75x110 cm	
	Ultrasound cover	
Patient preparation	3 sponges	
	5 gauze	
	ECG cable	
Central line catheter insertion	Ultrasound gel	
	3-way infusion ports	
	Syringe 10 mL	
	Scalpel	
	Needle 0.9x40 mm	
	Needle 0,7x30	
	5 compresses	
	Syringe 3 mL	
	Triple lumen catheter (TLC)	Triple lumen catheter (TLC)
	Nitinol guide wire	Nitinol guide wire
	Seldinger needle	Seldinger needle
	Plastic dilator	Plastic dilator
Central line fixation	TLC holder/ clip	TLC holder/ clip
	Suture thread with attached curved needle size 2–0, 75 cm	
	Needle driver	
	Adhesive bandage	

Prepackaged kit: prepackaged all-inclusive central line catheter insertion kit containing all of the necessary materials for insertion from preparation to cleanup. Standard kit: central line catheter insertion kit containing only the separately packaged catheter components. The remaining items were available from the materials cart.

### Study cohort

Novice residents and final year medical students were randomized into two equal groups using a random number list. To identify possible confounders, the following data were recorded for all participants: educational status (year of medical education, former studies in health care); prior central line catheter insertion experience for both manikins and patients (number of procedures); age; and sex. We excluded participants who had previously performed central line catheter insertion more than 15 times.

Both groups were assisted by first year nursing students with limited experience assisting with central line catheter insertion. Each nursing student assisted only once or twice during the study.

### Central line catheter kits

One group performed central line catheter insertion using the prepackaged kit and the other group used the standard kit with additional necessary items provided in the clinic’s stocked standard materials cart (see Table 1). The prepackaged kit contained almost all of the items necessary for the central line catheter insertion from preparation to cleanup, while the standard kit contained only the components of the central line catheter itself. This meant that the standard kit group had to decide which additional items were needed for the procedure and actively select them from the materials cart or ask the nurse for them at a later stage. We designed the prepackaged kit based on our internal departmental standards and safety policies. B. Braun Melsungen AG (Melsungen, Germany) manufactured and supplied both kits. The prepackaged kit was labeled to indicate that it did not include saline, lidocaine, sterile gloves, mask, cap, or blood gas syringe, because these materials were either not sterilizable (plastic containers or syringes, mask, cap) or dependent on the physician’s individual size (gloves). The standard kit is routinely used in our tertiary care hospital and contained a triple lumen catheter (TLC), a nitinol guide wire, a Seldinger needle, a plastic dilator and a TLC holder/clip. All other materials (including drapes, syringes, and gauze) had to be selected separately from the materials cart that is used for a variety of puncture procedures in our clinic (see Table 1), including ascites drainage and bone marrow aspiration. The materials cart contained the items used for these other procedures as well as the additional items needed for central line insertion when using the standard kit.

### Simulation manikin

The insertion procedure was performed on a central line manikin (SimuLab Corporation, Seattle, Washington, USA). The model was chosen because it is well established in teaching central line catheter insertion. It has a realistic anatomical jugular vein access and permits ultrasound guidance to identify both arterial and venous vessel tubes [19]. Over 150 punctures per silicon block are possible according to the manufacturer’s information (personal communication).

### Procedure evaluation by video assessment

The procedure was videotaped and the videos were analyzed by two reviewers using a checklist (Table 2). The raters were experienced physicians (internal medicine consultants) and clinical skills teachers at another university hospital. The camera was angled to focus on the central line catheter insertion team and the manikin, but also included the materials cart and preparation table to allow assessment of the complete insertion procedure, including breaches of aseptic technique. The reviewers had no information on the study design or the study question. The checklist (Table 2) included 55 steps [20]. We also assessed five quality indicators:

procedure duration (from the start of the preparations until the end of the cleanup process)

major technical mistakes (each deviation from the correct central line catheter insertion procedure that might have resulted in patient harm according to the rater’s judgment)

minor technical mistakes (each deviation from the correct central line catheter insertion procedure that might not have resulted in patient harm according to the rater’s judgment)

number of correctly performed steps according to the checklist (each step of the central line catheter insertion procedure that was performed in the correct order with the correct technique)

breaches of aseptic technique (each contact between sterile and unsterile material).

**Table 2:** Procedural checklist used by the video raters to evaluate performance

**Action**	**Correct**	**Minor technical error**	**Major technical error**	**Breaches in aseptic technique**
**Preparation of materials (26 Points):**				
Sterile gloves				
Sterile gown				
Cap				
Mask				
Disinfecting agent				
Sterile gauze				
Sterile compresses				
Local anesthetic				
3ml Syringe (for the anesthetic)				
Needle (for the anesthetic)				
Sterile drape				
Sterile fenestrated drape				
10 ml Syringe				
Distilled water (to simulate NaCl 0.9%)				
TLC				
Seldinger needle				
Guide wire				
Dilator				
Scalpel				
3-way ports				
Blood gas syringe				
TLC holder/ clip				
Suture thread				
Needle driver				
Adhesive bandage				
Sharps container				
**Patient preparation (6 Points):**				
Sterile use of ultrasound equipment, if applied to find the vein				
Hand washing/ sanitizing				
All of the following are worn: sterile gloves and gown, mask, cap				
Disinfection of the injection site				
Fenestrated drape is applied to injection site				
Injection of the local anesthetic (must aspirate before injection!)				
**Central line insertion (19 Points):**				
Both 10 mL syringes are filled with NaCl				
All 3 lumina of the catheter are flushed with NaCl				
All ports are blocked after the flush				
Repeat disinfection of the injection site (assistant)				
Insertion of Seldinger needle (30° angle)				
Patient is asked if he can still feel the needle				
The needle is inserted until the vein is punctured and blood can be drawn				
A sample for blood gas analysis is drawn				
Blood gas syringe is transferred to assistant				
Insertion of the guide wire				
Removal of the needle (the guide wire is secured in place)				
Skin incision along the guide wire				
Insertion of the dilator over the guide wire				
Insertion of the catheter over the guide wire				
The catheter is inserted through the skin only after the guide wire is secured				
Removal of the guide wire (the catheter is secured in place)				
Safe disposal of the guide wire (double knot/ sharps container)				
Blood is drawn from all 3 lumina				
All 3 lumina are flushed with NaCl				
**Securing the catheter and clean-up (4 Points):**				
TCL clip is attached to the catheter				
TLC clip is sutured in place				
The site of insertion is covered with an adhesive bandage				
All needles and the scalpel are safely disposed of (sharps container) and the work station is left clean				
**Sum (max. 55 Points)**				
**Miscellaneous:**				
Duration of the procedure				
Number of misunderstandings (Physician-Assistant)				
Number of questions from the assistants				

### Statistics

All data provided by the video raters were entered into a Microsoft ACCESS 2008 (Microsoft Corporation, Redmond, WA) database and subsequently analyzed using the JMP 8.0 software package (SAS Institute, Cary, NC, USA). We used Student’s t-test on normally distributed numerical data, the Wilcoxon test for non-normally distributed numerical data and the Chi^2^ test on parametric data. We used G*Power software (Erdfelder, Faul, & Buchner, 1996, Düsseldorf, Germany) for the power analysis, aiming for a power ≥ 0.80 based on the results of previous studies conducted by our research team [21]. A *p*-value of ≤ 0.05 was considered statistically significant. Interrater reliability was calculated using IBM SPSS Statistics Version 20 as an intraclass correlation coefficient with a two-way mixed-effects model (absolute agreement) (ICC (3,k)) according to Shrout and Fleiss’ definition [22].

### Ethical issues

The study protocol was reviewed and accepted by the local ethics committee, decision number 059/2011BO1. Study participation was voluntary. The results remained anonymous and were not used in any academic evaluations or assessments of the participants. All participants gave written informed consent and the study was performed in accordance with the declaration of Helsinki, revised form, Seoul 2008 [13].

## Results

### Power analysis

Power analysis revealed that at least 10 subjects for each group (prepackaged vs. standard kit) were required to detect an effect size “d” of 1.2 with a power of 80%, assuming a standard deviation of 5 (out of 55 checklist points) and normally distributed assessment scores in both groups. Because the test scores in the standard kit group were non-normally distributed in our study and the effect size was lower than estimated (0.94 vs. 1.2), the final power was 69%, despite each team including 15 participants with complete data sets in each group.

### Study cohort and randomization

Thirty physicians were randomized into two equal groups (n = 15). All applicants fulfilled the criteria of 15 or fewer previous central line catheter insertion procedures. Twenty-three (77%) had performed three or fewer procedures, resulting in an adequate target cohort for this study.

The study participants’ characteristics after randomization are summarized in Table 3. There were no differences between the prepackaged kit group and standard kit group with regard to sex, age, previous experience in central line catheter insertions and educational status (p > .12).

**Table 3:** Study participants’ characteristics

	**Prepackaged kit**	**Standard kit**	**P χ**^**2**^**, TT, Wilcoxon**
Gender	7 male, 8 female	11 male, 4 female	.13
Age	27.3 ± 2.5	27.3 ± 2.2	.93
Prior experience in central line catheter insertion on manikins (n = participants)	0: n = 13	0: n = 10	.36
	1-3: n = 2	1-3: n = 4	
	4-15: n = 0	4-15: n = 1	
Prior experience in central line catheter insertion on patients (n = participants)	0: n = 7	0: n = 7	.93
	1-3: n = 5	1-3: n = 6	
	4-15 : n = 3	4-15: n = 2	
Level of Education	6 final year medical students	8 final year medical students	.46
	9 novice residents	7 novice residents	

Results are displayed as mean ± SD.

### Procedure evaluation

**Figure 1:**
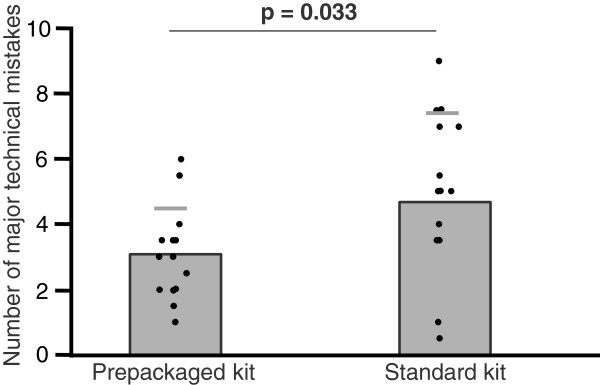
**Major technical mistakes.** Number of major technical mistakes in the prepackaged and standard kit group. Results are displayed as mean ± SD.

**Figure 2:**
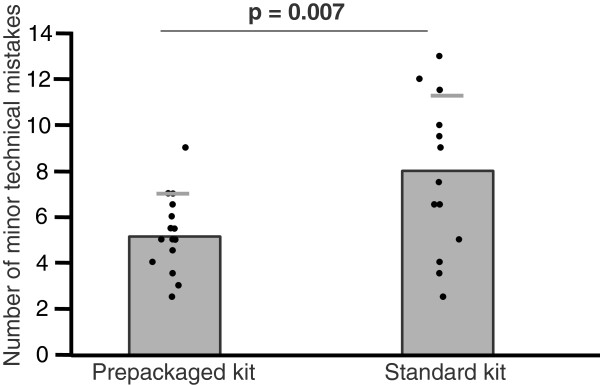
**Minor technical mistakes.** Number of minor technical mistakes in the prepackaged and standard kit group. Results are displayed as mean ± SD.

**Figure 3:**
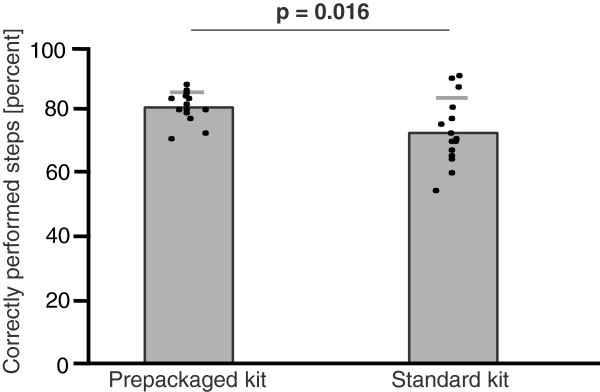
**Correctly performed steps.** Number of correctly performed steps in the prepackaged and standard kit group. Results are displayed as mean ± SD.

**Figure 4:**
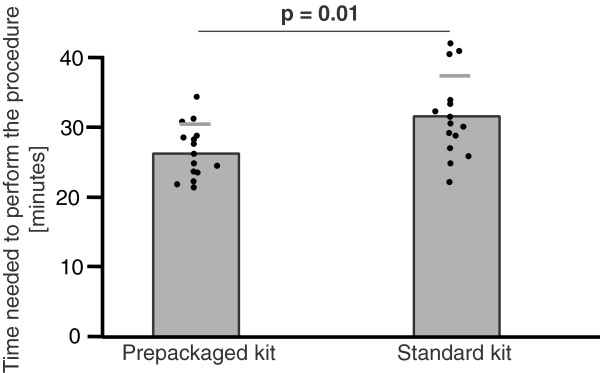
**Procedure duration.** Procedure duration in the prepackaged and standard kit group. Results are displayed as mean ± SD.

**Figure 5:**
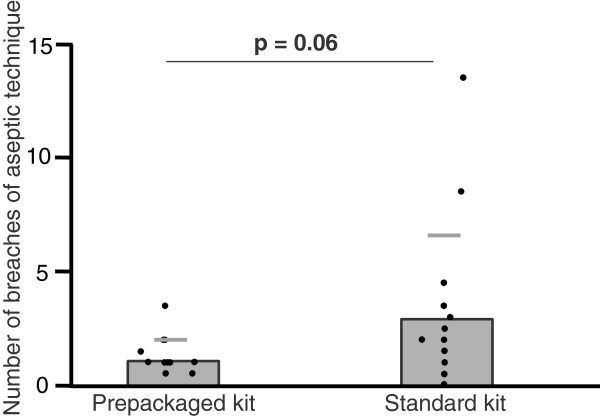
**Breaches in aseptic technique.** Number of breaches in aseptic technique in the prepackaged and standard kit group. Results are displayed as mean ± SD.

The prepackaged kit group made 35% fewer major mistakes (3.1 ± 1.4 vs. 4.8 ± 2.6, mean ± SD, p = .033; Figure 1) and 35% fewer minor mistakes (5.2 ± 1.7 vs. 8.0 ± 3.2, p = .007; Figure 2). With regard to the checklist, the prepackaged kit group adhered better to the procedural algorithm (83 ± 5% vs. 75 ± 11% of correctly performed steps, p = .016; Figure 3). The prepackaged kit group required less time to perform the procedure than the standard kit group (26:26 ± 3:50 min vs. 31:27 ± 5:57 min, p = .01; Figure 4). Although not statistically significant, there was a trend toward fewer breaches of aseptic technique in the prepackaged kit group (1.2 ± 0.8 vs. 3 ± 3.6, p = .06; Figure 5).

### Interrater reliability

The interrater reliability for the two blinded video raters calculated by interclass coefficient was .841.

## Discussion

Central line catheter insertion is a frequently used, complex, multistep procedure that produces a high cognitive load for novices. We used a worst-case scenario for our study: a novice trying to insert a central line catheter with assistance from an inexperienced nurse. We compared the effect of a prepackaged kit containing all of the possible necessary additional equipment with a standard kit containing only the central vein catheter and all other separately packaged components provided in a materials cart (see Table 1) for five quality indicators: procedure duration; adherence to the procedural algorithm (percentage of correctly performed steps); the number of major and minor technical mistakes; and the number of breaches of aseptic technique. A simulated setting was chosen to guarantee patient safety and to avoid any relevant variables that could interfere with the study question. We hypothesized that the use of a prepackaged kit would simplify the procedure in such a way that complications would be reduced [17]. Because the novices randomized to the prepackaged kit did not have to concentrate on the active selection of additional items required for the insertion procedure, the use of the prepackaged kit represented the split-attention principle, resulting in the reduction of cognitive load.

In four of the five categories, the novice residents and final year medical students who used the prepackaged kit outperformed those in the standard kit group. Putting most of the items required for the procedure into one prepackaged set was just one of the multistep interventions that have been shown to reduce the rate of central line catheter complications [10,18]. However, because the use of a prepackaged kit was not the only measure that was introduced in an attempt to lower the complication rate in previous studies, it was difficult to ascertain to what extent the prepackaged kit actually contributed to procedure safety.

We wanted to identify the potential beneficial effect of a prepackaged kit for three reasons:

1. Novices have been shown to have a higher complication rate than experts and need all the help they can get to minimize potential complications.

2. The use of prepackaged kits can be applied to other invasive procedures with a high cognitive load for novices, e.g., insertion of a chest tube or bone marrow aspiration.

3. The use of a prepackaged kit facilitates materials manipulation and allows for homogenous sterilization.

These advantages may outweigh the additional costs of prepackaging and other possible drawbacks such as material surplus (invariably, not all components are used) and a certain inflexibility because the same manufacturer must be used for the individual components.

Our study has several limitations. First, we standardized the procedure by using a manikin. Because we did not assess central line catheter insertion in actual patients, our results cannot be extrapolated. Second, despite having performed a pre-study power analysis, our study was ultimately underpowered and failed to detect a significant difference for one of the five quality indicators (number of breaches of aseptic technique). The category “breaches of aseptic technique” is only a surrogate parameter for a central line catheter bloodstream infection, as is non-adherence to the procedural algorithm for mechanical complications. A breach of aseptic technique and non-adherence to the procedural algorithm may or may not result in actual patient harm.

More research is needed to determine the effect of prepackaged kits on patient outcomes for other complex procedures and on factors that contribute to patient safety.

## Conclusions

The use of prepackaged kits may help reduce procedural mistakes in the central line catheter insertion procedure for novices.

## Competing interests

The authors declare that they have no competing interests.

## Authors’ contributions

YF made substantial contributions to the study design, performed the data acquisition and drafted the manuscript. NC made substantial contributions to the study design, helped analyze the data and drafted the manuscript. RW performed the statistical analysis and revised the manuscript critically. CN rated the videos and revised the manuscript critically. FL rated the videos and revised the manuscript critically. SZ made substantial contributions to the conception of the study and revised the manuscript critically. RR made substantial contributions to the conception of the study and revised the manuscript critically. PW designed the study, performed the data analysis and helped draft the manuscript. All authors read and approved the final manuscript.

## Pre-publication history

The pre-publication history for this paper can be accessed here:

http://www.biomedcentral.com/1472-6920/13/60/prepub
